# Incomplete Recruitment of Protective T Cells Is Associated with Trypanosoma cruzi Persistence in the Mouse Colon

**DOI:** 10.1128/iai.00382-21

**Published:** 2022-02-17

**Authors:** Alexander I. Ward, Michael D. Lewis, Martin C. Taylor, John M. Kelly

**Affiliations:** a Department of Infection Biology, London School of Hygiene and Tropical Medicine, London, United Kingdom; University of Pennsylvania

**Keywords:** *Trypanosoma cruzi*, Chagas disease, chronic persistence, murine imaging, colon, T cell recruitment

## Abstract

Trypanosoma cruzi is the etiological agent of Chagas disease. Following T cell-mediated suppression of acute-phase infection, this intracellular eukaryotic pathogen persists long-term in a limited subset of tissues at extremely low levels. The reasons for this tissue-specific chronicity are not understood. Using a dual bioluminescent-fluorescent reporter strain and highly sensitive tissue imaging that allows experimental infections to be monitored at single-cell resolution, we undertook a systematic analysis of the immunological microenvironments of rare parasitized cells in the mouse colon, a key site of persistence. We demonstrate that incomplete recruitment of T cells to a subset of colonic infection foci permits the occurrence of repeated cycles of intracellular parasite replication and differentiation to motile trypomastigotes at a frequency sufficient to perpetuate chronic infections. The lifelong persistence of parasites in this tissue site continues despite the presence, at a systemic level, of a highly effective T cell response. Overcoming this low-level dynamic host-parasite equilibrium represents a major challenge for vaccine development.

## INTRODUCTION

The insect-transmitted protozoan parasite Trypanosoma cruzi is the causative agent of Chagas disease and infects 5 to 7 million people in Latin America (1). Despite decades of effort, only limited progress has been made in developing a vaccine, and doubts remain about the feasibility of vaccination as a method for disease control (2, 3). In humans, T. cruzi infection passes through an acute stage that lasts 2 to 8 weeks, during which parasitemia is readily detectable, although symptoms are generally mild and nonspecific. With the induction of the adaptive immune response, in which CD8^+^ gamma interferon-positive (IFN-γ^+^) T cells play a key role (4, 5), there is a significant reduction in the parasite burden. However, sterile clearance is not achieved, and parasites persist as a chronic lifelong infection. One-third of those infected with T. cruzi eventually develop Chagasic pathology, although symptoms can take decades to become apparent. Cardiomyopathy is the most common clinical outcome (6–8), followed by digestive tract dysfunction and megasyndromes, which are reported in about 10% of infected individuals, often in parallel with cardiac disease.

Although the innate immune system is able to detect the parasite (9, 10), there is a delay in the subsequent induction of an adaptive response relative to other pathogens (5, 11). This, together with a high rate of parasite division (12) and broad cell type tropism, allows T. cruzi to disseminate widely during the acute stage, with most organs and tissues becoming highly infected (13). The CD8^+^ T cell response, which predominantly targets a subset of immunodominant epitopes in members of the hypervariable *trans*-sialidase surface antigen family (14, 15), is critical for controlling the acute-stage infection in mice, in combination with antibody-mediated responses. The parasite burden is reduced by 2 to 3 orders of magnitude as the disease transitions to a chronic dynamic equilibrium (13). Understanding why the immune system then fails to eliminate the remaining parasites is a central question in Chagas disease research. This information is crucial to underpin rational vaccine design and immunotherapeutic interventions.

Because of the complexity and long-term nature of Chagas disease in humans, mice have been important experimental models for research on interactions between parasite and host. They display an infection profile similar to that in humans, exhibit chronic cardiac pathology, and are widely used in drug and vaccine development (16). Bioluminescence imaging studies have revealed that the gastrointestinal (GI) tract is a major parasite reservoir during chronic infections and that the degree of containment to this region is determined by both host and parasite genetics (13, 17). Parasites are also frequently detectable in the skin, and in some mouse models, such as C3H/HeN, skeletal muscle can be an important site of persistence (4, 18). In the colon, the most frequently infected cells are myocytes located in the gut wall. However, the extent of infection is low, and in many cases, this entire organ contains only a few hundred parasites, concentrated in a small number of host cells (18). After transition to the chronic stage, T. cruzi also exhibits a reduced proliferation rate, although the cycle of replication, host cell lysis, and reinfection appears to continue (12). Evidence for a form of dormancy in T. cruzi has been reported (19); however, whether this is analogous to dormant/quiescent life cycle stages observed in other parasites, such as Toxoplasma gondii bradyzoites and Plasmodium vivax hypnozoites (20), remains to be established.

Multiple studies have shown that experimental T. cruzi vaccines have protective efficacy and can reduce both parasitemia and disease severity (21–26). However, evidence for complete parasite elimination after challenge is lacking. In contrast, drug-cured infections can confer long-lasting protection against rechallenge with a homologous parasite strain (3), although sterile protection was achieved in only ∼50% of animals. Rechallenge with a heterologous strain did not result in sterile protection, although there was a >99% reduction in the peak acute-stage parasite burden. All drug-cured animals that displayed reinfection transitioned to the canonical chronic-stage equilibrium and organ distribution, without passing through an elevated acute-stage parasitemia. Once established in permissive sites, such as the GI tract, parasites appear to survive the systemic T. cruzi-specific IFN-γ^+^ T cell response generated by the primary challenge. In the absence of information on the immunological microenvironment of these persistent parasites, the reasons for this are unclear. Resolving this question will have a major strategic impact on the development of an effective vaccine.

Progress in this area has been limited by technical difficulties in locating and analyzing the rare infection foci in permissive tissue sites, such as the colon. Here, we describe the application of a T. cruzi bioluminescent-fluorescent dual-reporter strain and enhanced tissue processing and imaging procedures that allowed us to show that incomplete homing of leukocytes, including T cells, to foci of intracellular infection is associated with the ability of the parasite to persist in the colon.

## RESULTS

### Cellular immunity suppresses the colonic parasite load during chronic T. cruzi infection.

**FIG 1:**
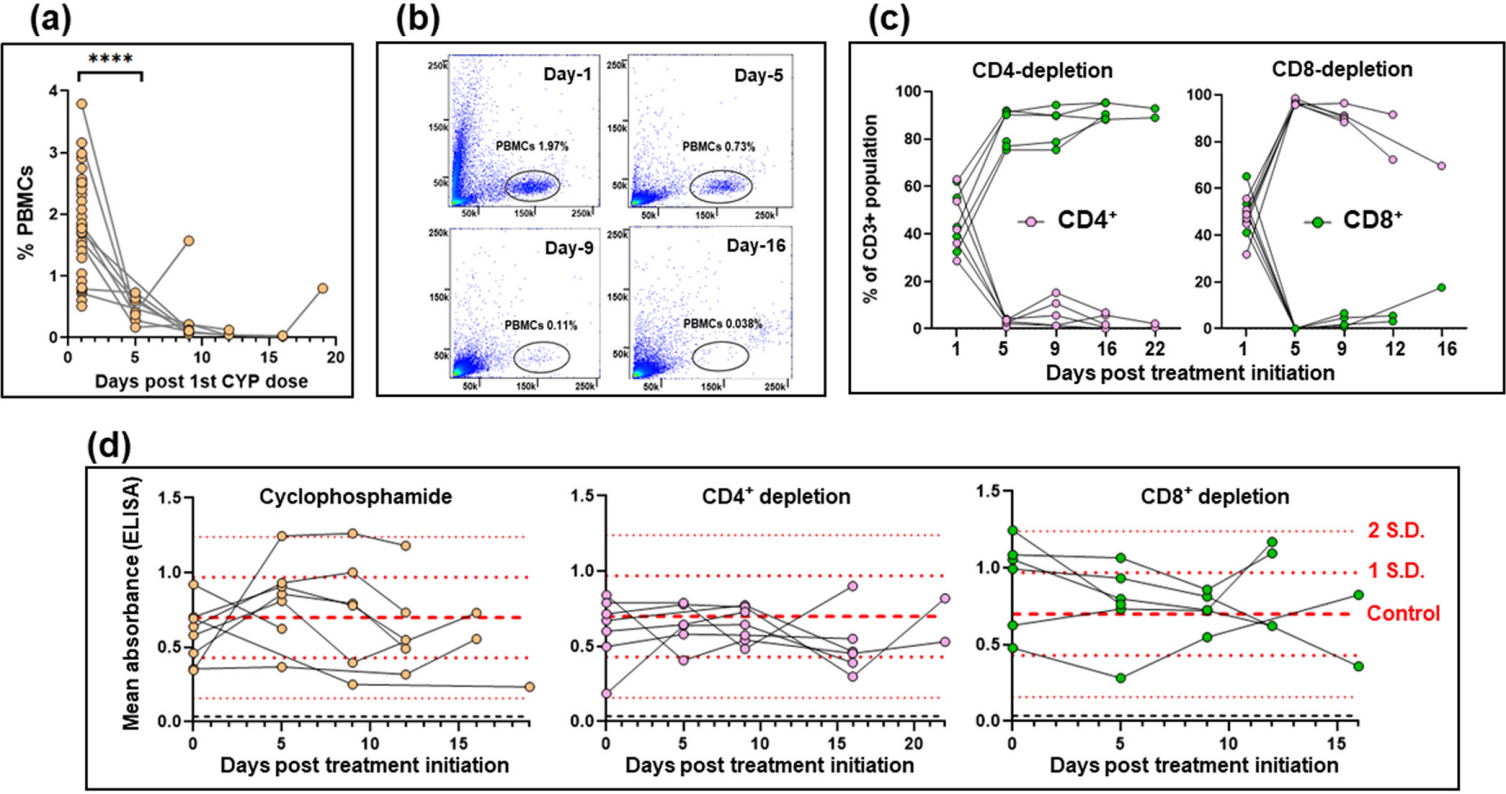
Suppression of cellular immunity in mice chronically infected with T. cruzi. (a) C3H/HeN mice chronically infected (>100 days) with T. cruzi CL Luc::mNeon (*n* = 6) were immunosuppressed by i.p. inoculation with cyclophosphamide (CYP; 200 mg/kg) at 4-day intervals, up to a maximum of 3 injections (Materials and Methods). The percentage of events recorded as peripheral blood mononuclear cells (PBMCs) at different time points after the initiation of treatment for individual mice are shown. Also included in the day 1 values are additional data points (*n* = 24) from immunocompetent chronically infected mice. ****, *P* < 0.0001. (b) Flow cytometry plots showing the loss of detectable events in the PBMC gate (black oval) over the course of cyclophosphamide treatment (see also Fig. S2). PBMCs were identified based on the spectral forward (*y* axis) and side (*x* axis) scatter. (c) Effective depletion of T cell subsets by treatment of mice with specific anti-CD4 or anti-CD8 antibodies (Materials and Methods). The graphs show the CD4^+^ and CD8^+^ flow cytometry events of individual mice as a percentage of the total CD3^+^ population over the treatment periods. (d) ELISA mean absorbance readings (using anti-mouse IgG secondary antibody) for serum from chronically infected mice that had been treated with cyclophosphamide or treated with anti-CD4 or anti-CD8 antibodies. Microtiter plates containing T. cruzi trypomastigote lysates were prepared as described in Materials and Methods. Dashed red lines identify the mean, ±1 SD, and ±2 SD values, determined from immunocompetent chronic-stage controls (*n* = 28). One of the anti-CD8 antibody-treated mice died between days 5 and 9 and was excluded from subsequent analysis.

Myocytes in the colonic gut wall are an important site of T. cruzi persistence in murine models of chronic Chagas disease. However, infected host cells are extremely rare and unevenly distributed (18), and their immunological microenvironment has not been systematically investigated. To assess the role of the immune response in controlling infection at a cellular level in this tissue compartment, we infected C3H/HeN mice with T. cruzi CL Luc::mNeon, a parasite line that constitutively expresses a bioluminescent-fluorescent fusion protein (27). This reporter strain can be used in combination with *ex vivo* imaging and confocal microscopy of colonic wall whole mounts to detect infection foci at single-cell resolution (Materials and Methods). When infections reached the chronic stage (>100 days postinfection), one cohort of mice was immunosuppressed with cyclophosphamide, an alkylating agent which is generally suppressive of leukocyte populations, including both innate cells and T cells (28), and which has been widely used to drive the reactivation of low-level T. cruzi infections in experimental settings (29–32). Cyclophosphamide itself has no growth-promoting effect on intracellular amastigotes (see Fig. S1 in the supplemental material). Treatment led to a major reduction in peripheral blood mononuclear cells (PBMCs) within 5 to 10 days (Fig. 1a and b; Fig. S2). In parallel, other groups of mice were subjected to antibody-mediated depletion of the circulating CD4^+^ or CD8^+^ T cell populations (Materials and Methods). This was achieved, with high specificity, in a similar time scale (Fig. 1c; Fig. S2). Levels of circulating anti-T. cruzi serum antibody were not significantly altered by cyclophosphamide treatment or by depletion of the CD4^+^ or CD8^+^ T cell subtypes (Fig. 1d).

**FIG 2:**
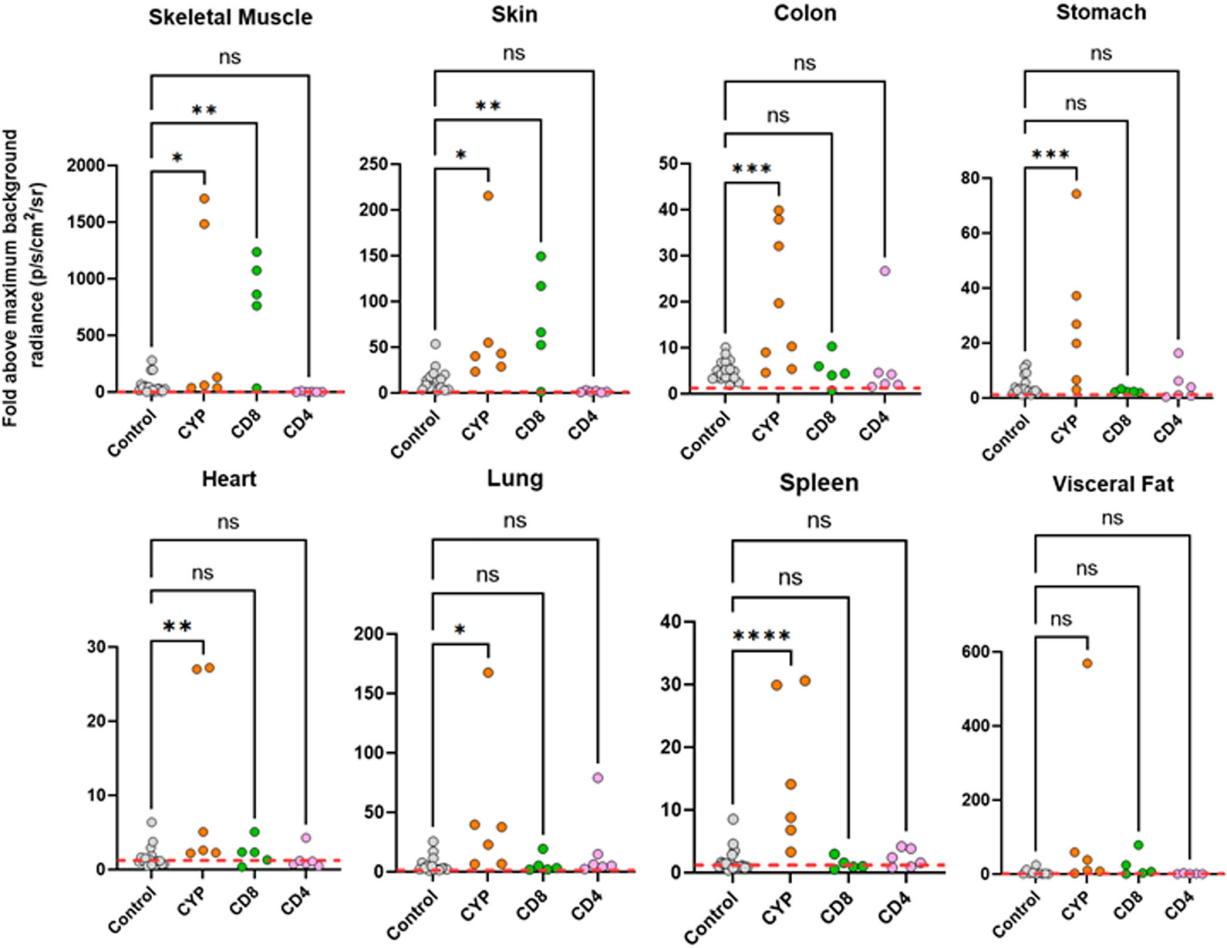
Fold change in radiance (photons/s/cm^2^/sr) established by *ex vivo* bioluminescence imaging of internal tissues and organs from C3H/HeN mice chronically infected with T. cruzi (control) and after treatment with cyclophosphamide (CYP), anti-CD4, or anti-CD8 antibodies, as indicated (Materials and Methods). Infection intensities were determined using LivingImage software to draw individual regions of interest around each organ and tissue sample (17). Data from infected mice were normalized to account for variations in background radiances of different tissue types by using matching tissues from uninfected controls to establish the fold change. The maximal value from the uninfected organs was used. The dashed line indicates the detection threshold, equal to the mean +2 SD of the bioluminescence background derived from the fold change between empty regions of interest in tissue from age-matched uninfected mice and empty regions from chronically infected animals. Control data points also include values from additional immunocompetent chronically infected mice (*n* = 17) (18). Means were compared with a one-way ANOVA with *post hoc* Dunnett’s pairwise comparisons test. *, *P* < 0.05; **, *P* < 0.01; ***, *P* < 0.001; ****, *P* < 0.0001; ns, not significant.

**FIG 3:**
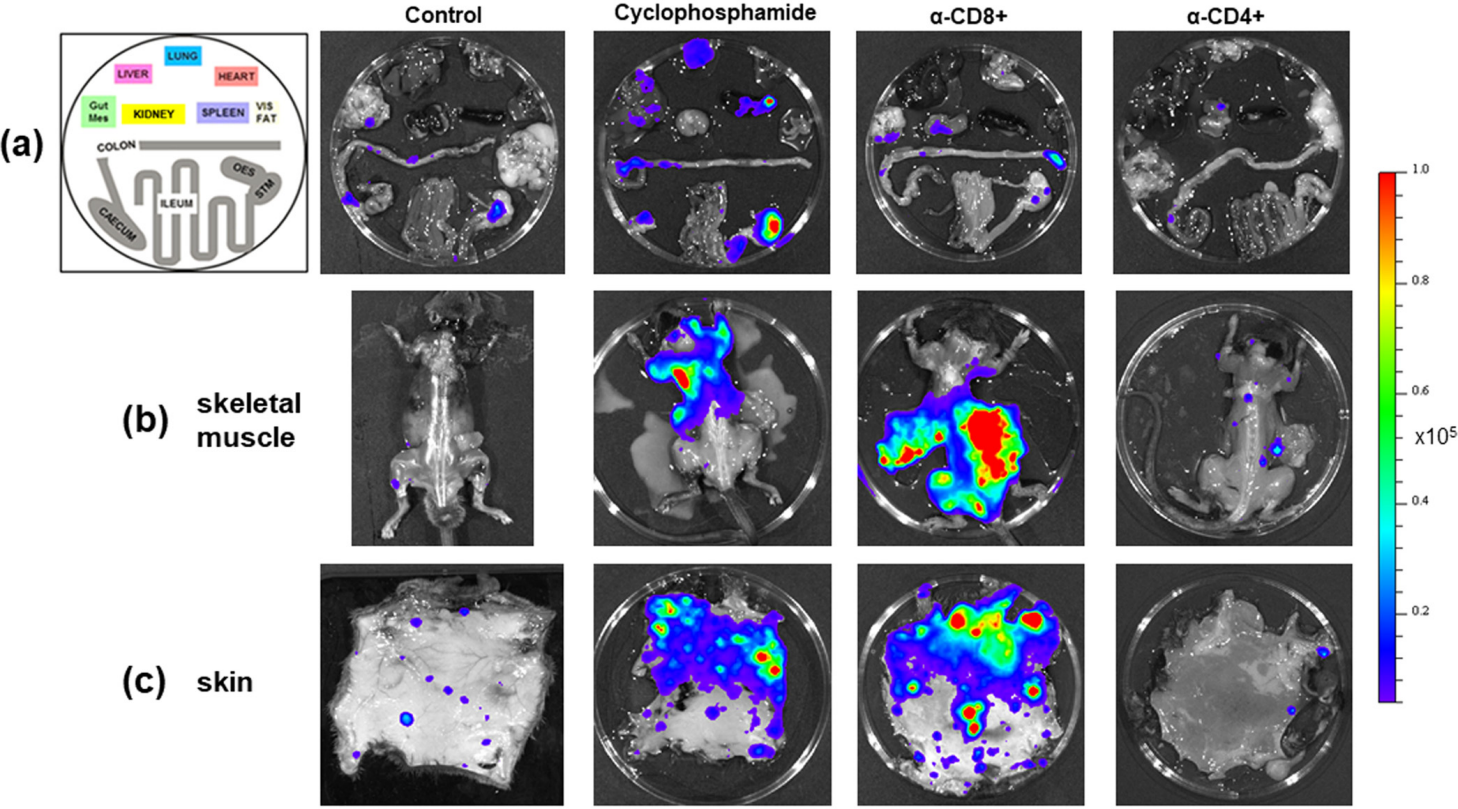
Tissue-specific impact of T cell depletion on parasite burden. C3H/HeN mice chronically infected with T. cruzi CL Luc::mNeon were treated with cyclophosphamide, anti-CD4, or anti-CD8 antibodies as outlined in the legend to Fig. 1. Sixteen days after treatment initiation, organs and tissues were examined by *ex vivo* imaging (52) (Materials and Methods). (a) Representative bioluminescence images of internal organs from treated mice arranged as shown on the left. Mes, mesentery; Vis, visceral (fat); OES, oesophagus; STM, stomach. (b) Dorsal bioluminescence images following removal of internal organs, fur, skin, and major adipose depots (Materials and Methods). (c) *Ex vivo* bioluminescence imaging of skin (adipose tissue removed). Radiance (photons/s/cm^2^/sr) is on a linear-scale pseudocolor heat map. The heat map image of skeletal bioluminescence after treatment with anti-CD8 antibodies is shown at an increased minimum and maximum radiance (1 × 10^4^ to 1 × 10^6^) to avoid saturation of the image. The complete radiance data set is shown in Fig. 2.

Examination of mouse tissue and organs by *ex vivo* bioluminescence imaging (13, 17) revealed that cyclophosphamide-induced immunosuppression had resulted in a widespread increase in the intensity of infection (Fig. 2 and 3), including in the skin, skeletal muscle, GI tract, and heart. With CD8^+^ depletion, skeletal muscle and the skin were the only tissue sites where we observed a significant enhancement in the level of infection, with increases greater than 1 order of magnitude in some instances. However, there was a wide variation in the parasite burden between mice (Fig. 2 and 3), reflecting the dynamic nature of chronic disease infections (13). In the internal organs, including the GI tract, there was no significant increase in infection as a result of CD8^+^ T cell depletion, at least within the time frame of the experiment. CD4^+^ T cell depletion did not promote a relapse in any of the organs or tissue sites examined (Fig. 2 and 3).

**FIG 4:**
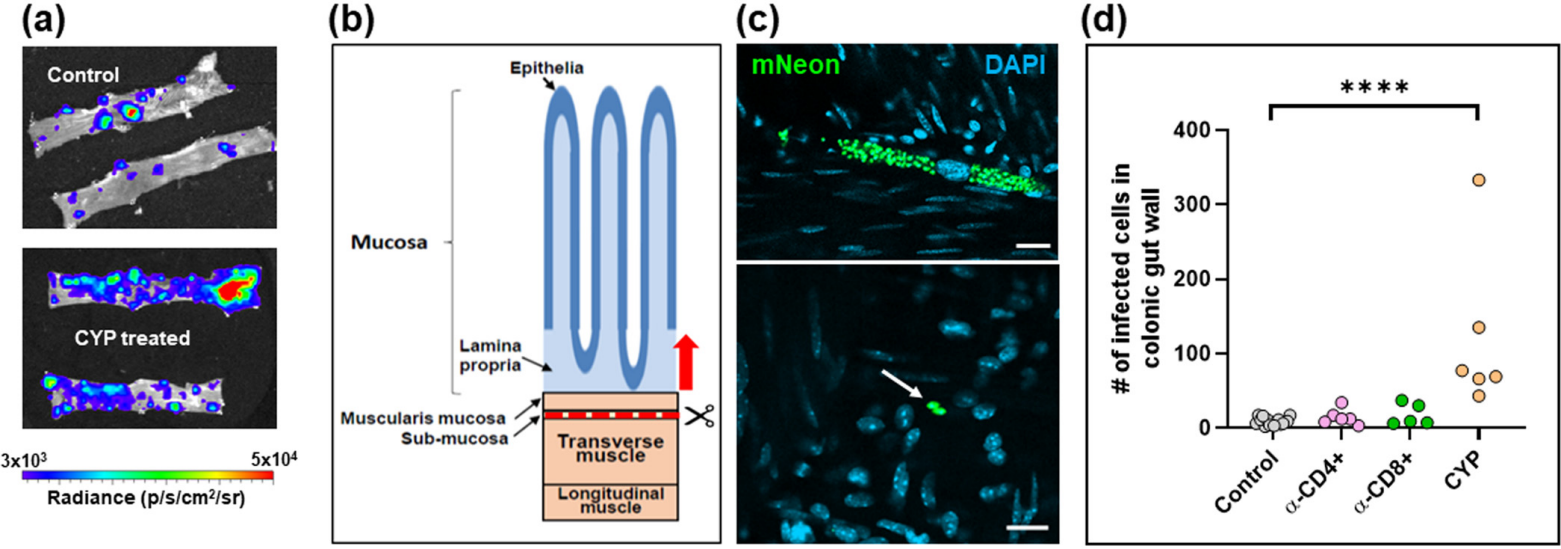
Control of parasites in the colons of chronically infected mice is lost on suppression of cellular immunity. (a) Colon sections from C3H/HeN mice chronically infected with T. cruzi CL-Luc::mNeon were pinned luminal side up and examined by *ex vivo* bioluminescence imaging. Radiance (photons/s/cm^2^/sr) is on a linear-scale pseudocolor heat map. (Top) Colonic sections from nontreated infected mice; (bottom) section from mice immunosuppressed by cyclophosphamide (CYP) treatment (Materials and Methods). (b) Schematic highlighting the distinct layers of the GI tract. The dashed red line and arrow indicate the position above which tissue can be peeled off to leave the external colonic wall layers (18). (c) External gut wall whole mounts were examined in their entirety at a 3-dimensional level by confocal microscopy. Examples of parasite-infected cells in immunocompetent mice and their locations, detected by green fluorescence (mNeon). DAPI staining (blue) identifies host cell nuclei. Bars = 20 μm. (d) Total number of parasitized cells counted in each whole mounted colonic gut wall for the control and the immune-depleted groups. Each dot represents a single mouse, with the colons examined 12 to 22 days after treatment initiation (Fig. 1d). ****, *P* ≤ 0.0001. Differences between control values and those obtained from mice that had been treated with anti-CD4 and anti-CD8 antibodies were nonsignificant.

To further investigate the effect of perturbing the immune system, we undertook detailed confocal microscopy analysis of external gut walls after removal of the mucosal layer (Materials and Methods). This technique allows systematic assessment of the full length and thickness of the longitudinal and circular smooth muscle layers of the colon at a single-cell level (18). Consistent with the *ex vivo* imaging data (Fig. 3), this revealed that cyclophosphamide treatment had resulted in a significant increase in the number of parasite-infected cells (Fig. 4). Furthermore, in the absence of PBMCs (Fig. 1a), it is implicit from the resulting parasite dissemination that the circulating serum antibodies are unable to maintain suppression of the infection at this and other sites during the chronic stage (Fig. 2 and 3). However, specific depletion of either the CD4^+^ or the CD8^+^ T cell repertoires (Fig. 1c), by themselves, did not have a significant effect on the number of infected cells in the colonic gut wall (Fig. 4d).

### Parasites persisting in the colon can induce effective localized T cell recruitment.

**FIG 5:**
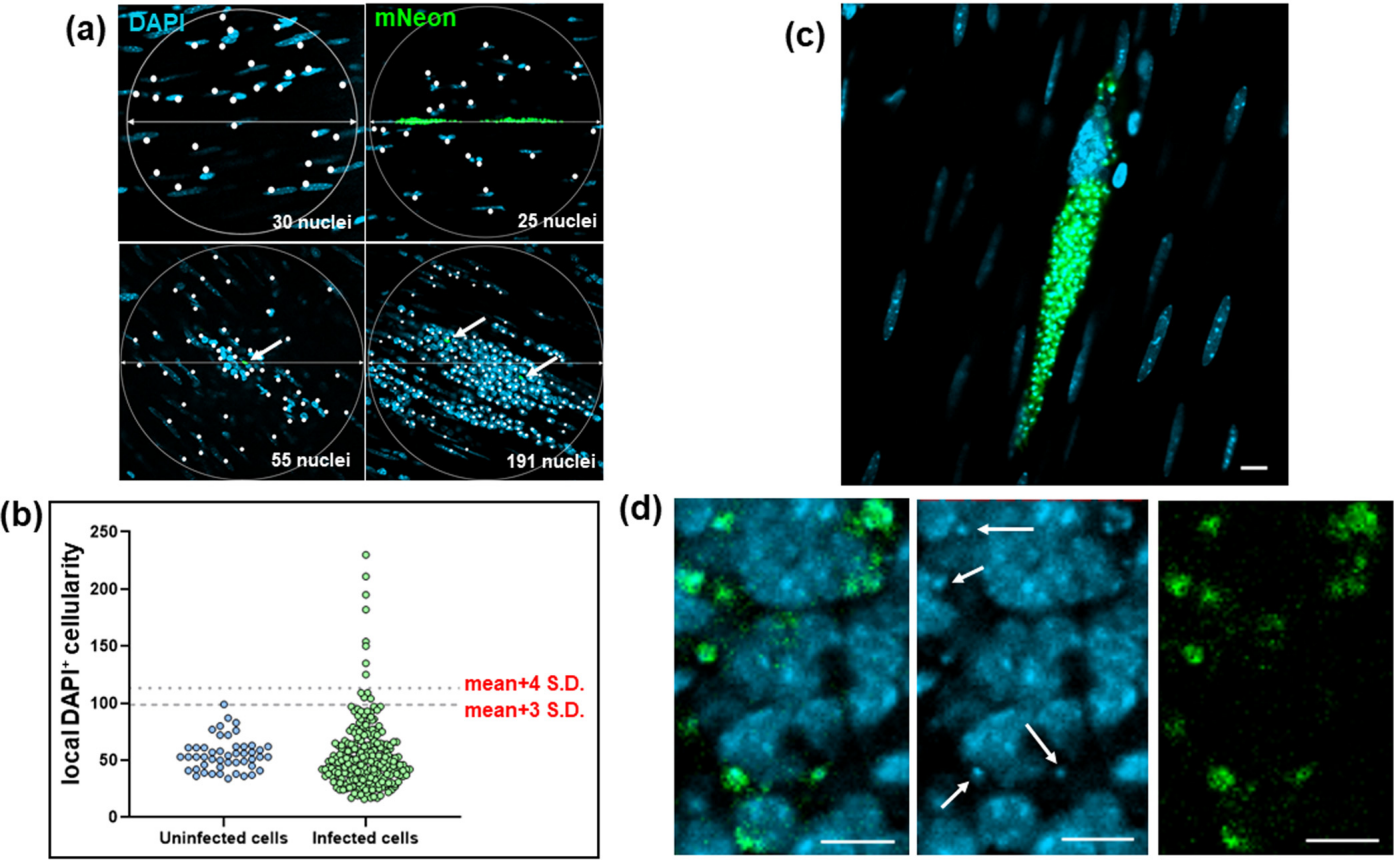
Defining the localized cellularity of T. cruzi-infected host cells in the colonic gut wall. (a) Images of whole mounted colonic gut wall from C3H/HeN mice chronically infected with T. cruzi CL-Luc::mNeon (Materials and Methods). When infection foci were identified, 200-μm-diameter circles were drawn centered on each parasite cluster, or nest. Circles were placed by centering on randomly selected cells in the case of noninfected age-matched controls (top left). DAPI-stained nuclei (blue) that fell within this disc (highlighted by white dots) were counted as a measure of cellularity. Intracellular parasites can be identified by green fluorescence. These are indicated by white arrows in the lower images. (b) Background cellularity around randomly selected cells (*n* = 48) on whole mounted colonic gut walls from naive age-matched C3H/HeN mice was established as described above. With tissue from chronically infected mice, localized cellularity was calculated using circles centered on parasite foci (green) (*n* = 247). Individual values are indicated by blue (noninfected) and green (infected) dots. The dashed lines indicate 3 SD and 4 SD above the background mean. (c) An infected myocyte where the local cellularity is equivalent to the background level and the intracellular amastigotes (green) are structurally intact. (d) Zoomed-in image of an intense cellular infiltrate (nuclei; blue) in which the T. cruzi parasites (green) display an irregular and diffuse morphology. Parasite DNA is identifiable as small discrete DAPI-stained spheres throughout this inflammatory focus (examples indicated by white arrows). Bars = 20 μm.

At any one time, the majority of the parasite population that persists in the colon is found in a small number of infected cells that typically contain several hundred replicating amastigotes or, occasionally, differentiated nondividing trypomastigotes (12). The remainder of the population is more widely distributed, with considerably lower numbers of parasites per infected cell. To better understand the process of long-term parasite survival, we investigated the cellular microenvironment of persistent infection foci. When infections had advanced to the chronic stage, peeled colonic wall whole mounts were examined by confocal microscopy (Materials and Methods) and compared to those of naive, age-matched control mice. In the tissue from noninfected mice, using DAPI (4′,6-diamidino-2-phenylindole) staining to highlight nuclei, an average of 55 host cells were identified in 200-μm-diameter circles positioned around randomly selected nuclei within the whole mounted gut wall (Fig. 5a). Most cells had elongated nuclei typical of smooth muscle myocytes. In the infected group, parasitized cells were identified by green fluorescence (Materials and Methods). Scanning revealed that total cellularity in the immediate locality of infection foci was similar in most cases to that in noninfected colonic tissue; the cellularity of 95% of infection foci was within 3 standard deviations (SD) of the background mean, compared with 98% around randomly selected cells from naive control regions (Fig. 5a and b). However, on occasion there was evidence of highly localized cellular infiltration, with 3.4% of infection foci surrounded by a local cellularity that was >4 SD above the background mean. Within these intense infiltrates, host cells with more rounded nuclei, typical of lymphocytes, predominated. In contrast to the majority of parasitized cells that had not triggered a detectable localized response (Fig. 5c), amastigotes in these inflammatory infiltrates frequently displayed an irregular morphology suggestive of immune-mediated damage, as judged by the diffuse pattern of green fluorescence (compare Fig. 5c and d).

**FIG 6:**
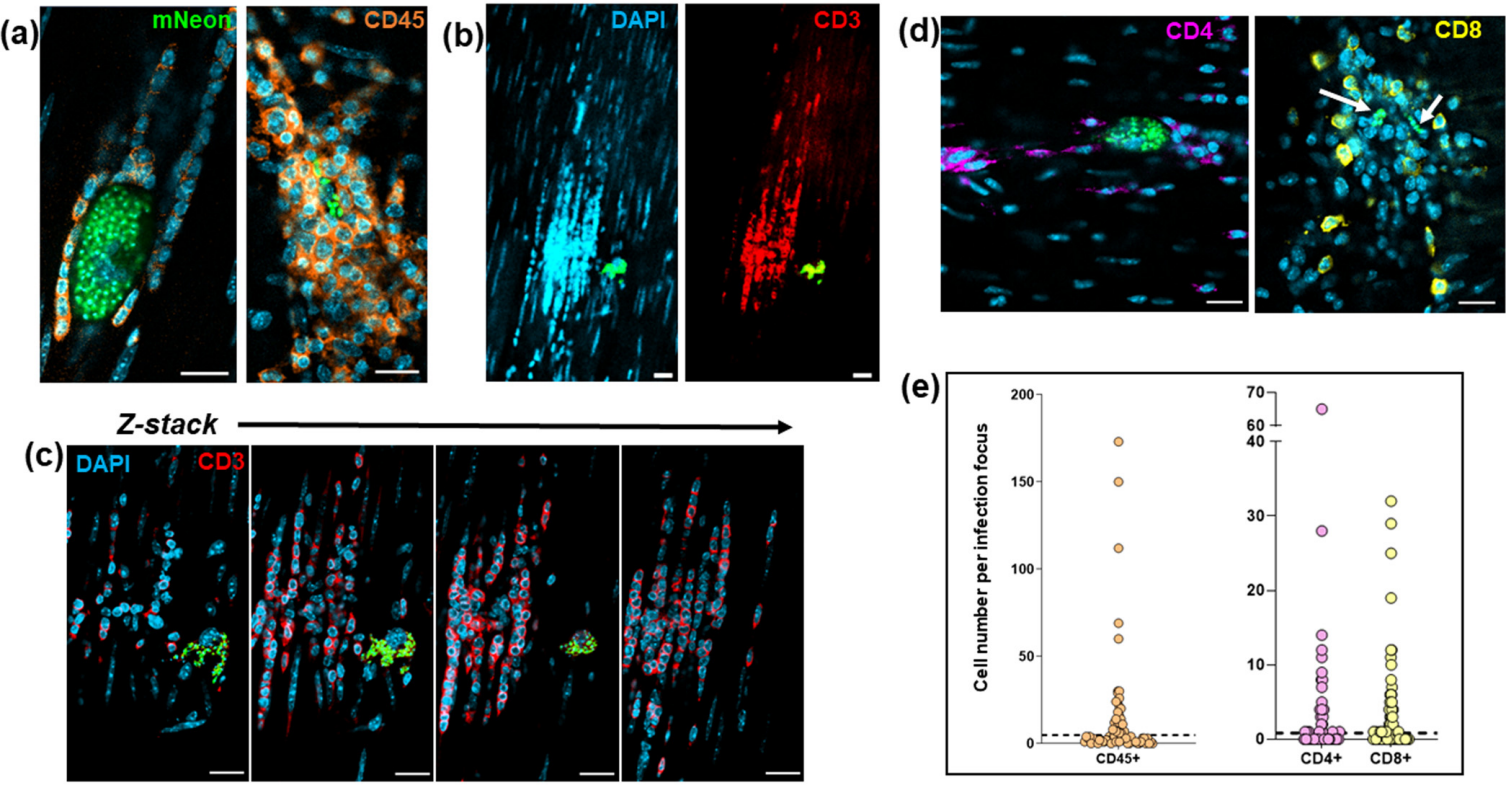
T cells are major constituents of the leukocyte population recruited to chronic-stage infection foci. (a) Confocal images of colonic gut wall sections from chronically infected mice (Materials and Methods). Rare infection foci were identified by mNeonGreen fluorescence (parasites) after exhaustive searching of whole mounted gut walls. Staining with anti-CD45 (orange) reveals that hematopoietic cells constitute the vast majority of the infiltrate population. Host cell nuclei were identified by DAPI staining (blue). (b) Anti-CD3 staining of cellular infiltrates shows that T cells constitute a majority of the population. Blue, host cell nuclei; red, CD3 staining; green, parasite fluorescence. (c) Serial Z-stack imaging (Materials and Methods) through the same cellular infiltrate as in panel b, showing selected sections through the infiltrate. (d) Histological sections containing cellular infiltrates and associated infection foci (parasites, green; indicated by white arrows in right-hand image) stained with either anti-CD4 (purple) or anti-CD8 (yellow) antibodies. Bars = 20 μm. (e) Whole mounts containing infection foci were stained with anti-CD45, anti-CD4, or anti-CD8 antibodies and the number of positive host cells in the immediate vicinity (314-μm^3^ volume) was determined by serial Z-stack confocal imaging. Each dot corresponds to a single infection focus. The horizontal dashed line is 3× above the SD of the mean background level in noninfected tissue. In the case of anti-CD45 staining, none of the 50 tissue regions examined from noninfected mice contained numbers of CD45^+^ cells above this value. Of infection foci identified by CD45, CD4, and CD8 staining, 41%, 45%, and 42%, respectively, were above this cutoff.

We investigated the nature of these cellular infiltrates, by staining colonic gut wall whole mounts from chronically infected mice with specific immune cell markers (Materials and Methods). This revealed, as expected, that leukocytes (identified by anti-CD45 antibodies) constituted close to 100% of the infiltrate population (Fig. 6a). A major proportion of the recruited cells were also positive when stained with anti-CD3 antibodies, specific markers for the T-cell receptor complex (Fig. 6b and c), with both CD4^+^ and CD8^+^ T cells represented within this population (Fig. 6d). To assess the local density of stained immune cells, we examined 200-μm-diameter circular tissue sections centered on each infection focus using Z-stack confocal microscopy. A series of imaged sections starting 5 μm above and 5 μm below the center of the parasite nest (a total volume of 314 μm^3^) were generated, and the number of stained cells in the infection microenvironment determined in 3 dimensions (Fig. S3). In sections of colonic smooth muscle from noninfected mice, leukocytes were dispersed and rare, with an average of ∼1 CD45^+^ cell per 314 μm^3^, although they were more numerous in the submucosal tissue (Fig. S4). Using a cutoff value of 3 SD above the respective background level, 40 to 45% of infection foci displayed evidence of leukocyte infiltration (Fig. 6e). Therefore, despite being a site of parasite persistence, dynamic homing of leukocytes, including T cells, to parasitized cells in the murine colon is a characteristic of chronic-stage infection, although at any one point in time, not all parasite nests will have triggered this type of recruitment response. Given the “snapshot” nature of imaging, our data therefore suggest that in the majority of cases, a likely outcome of colonic cell invasion will be infiltration of leukocytes prior to completion of the T. cruzi intracellular cycle and the presumptive destruction of the parasites (Fig. 5d).

### Incomplete homing of protective T cells allows a subset of intracellular colonic infections to complete their replication cycle.

**FIG 7:**
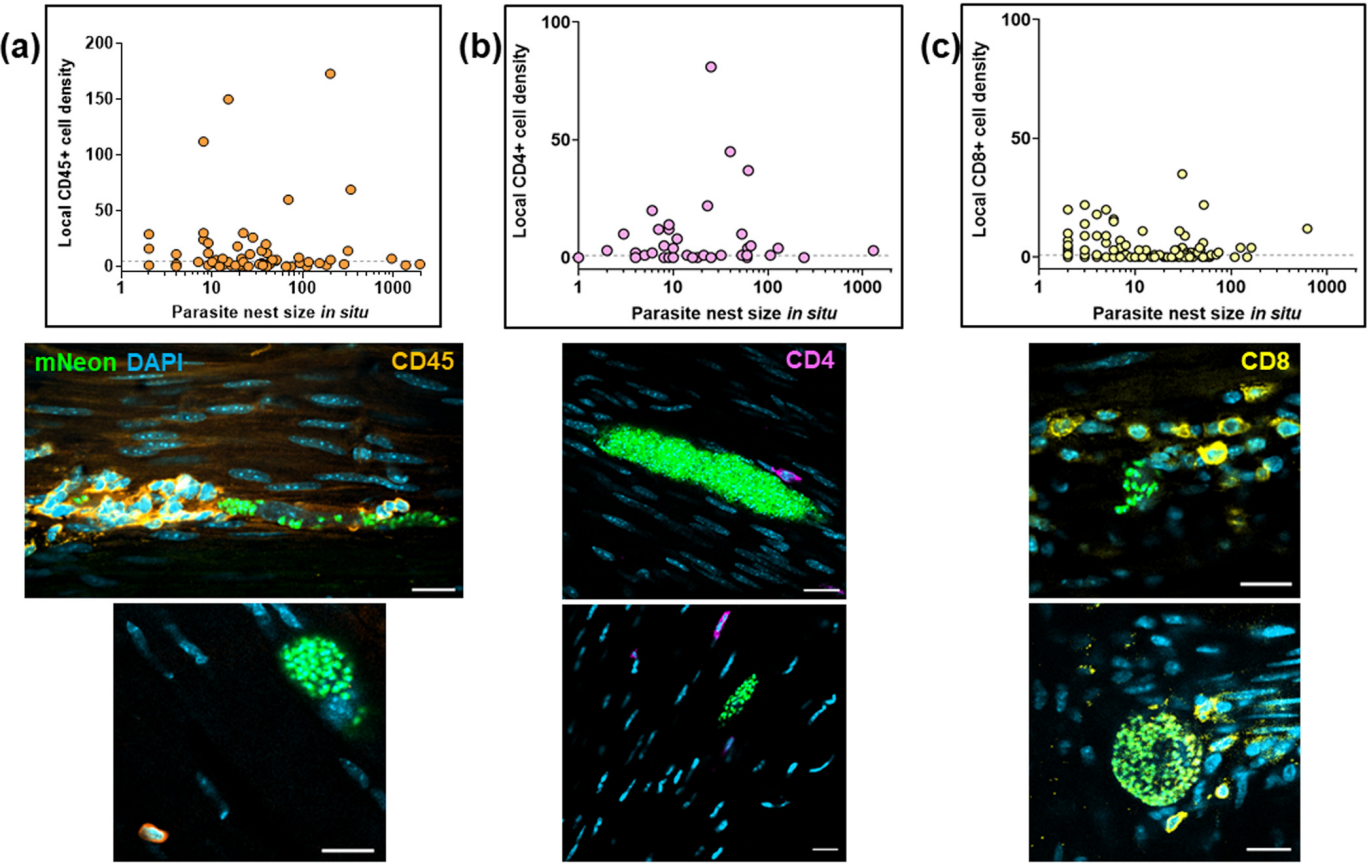
Lack of correlation between intracellular parasite load and localized T cell infiltration during chronic infections. (a) Comparison of the parasite numbers in infected colonic gut wall cells with the local leukocyte cell density. Infection foci were identified in whole mounts of colonic tissue, which were then stained with anti-CD45 antibody (Materials and Methods). The parasite and cell numbers in a tissue volume of 314 μm^3^ were determined using serial Z-stack imaging, with leukocytes identified by orange staining and parasites by green fluorescence. The horizontal dashed line is 3 SD above the mean background level in noninfected tissue. Each dot identifies a single infection focus, with tissue samples derived from 6 mice (71 infection foci). The confocal images show representative infection foci used to generate the data and illustrate the various extents of leukocyte infiltration. (b) Similar analysis of infection foci using anti-CD4 staining (purple). Tissue samples were derived from 3 mice (54 infection foci). (c) Analysis of infection foci using anti-CD8 staining (yellow). Tissue derived from 4 mice (116 infection foci).

Evidence indicates that T. cruzi rarely occupies individual colonic myocytes for extended periods (>2 weeks) (12), suggesting either that parasites are efficiently eliminated by the immune response or that they complete a cycle of replication, trypomastigogenesis, and host cell lysis within this period. In addition, there is considerable variation in the level of infection within individual colonic cells at any one time, with parasite numbers that can range from 1 to >1,000 (12). We therefore investigated whether the immune response induced against infected cells increased in line with the intracellular parasite burden. When the levels of infiltrating leukocytes in the local environment of infected cells were compared with the number of intracellular T. cruzi parasites, we found no apparent correlation (Fig. 7a to c). This was the case irrespective of whether anti-CD45, anti-CD4, or anti-CD8 antibodies were used to assess the nature of the cellular infiltrate. It is implicit, therefore, that the elapsed duration of an individual intracellular infection, as inferred from the extent of parasite proliferation, is not a determinant of the likelihood of detection and leukocyte homing to that site.

**FIG 8:**
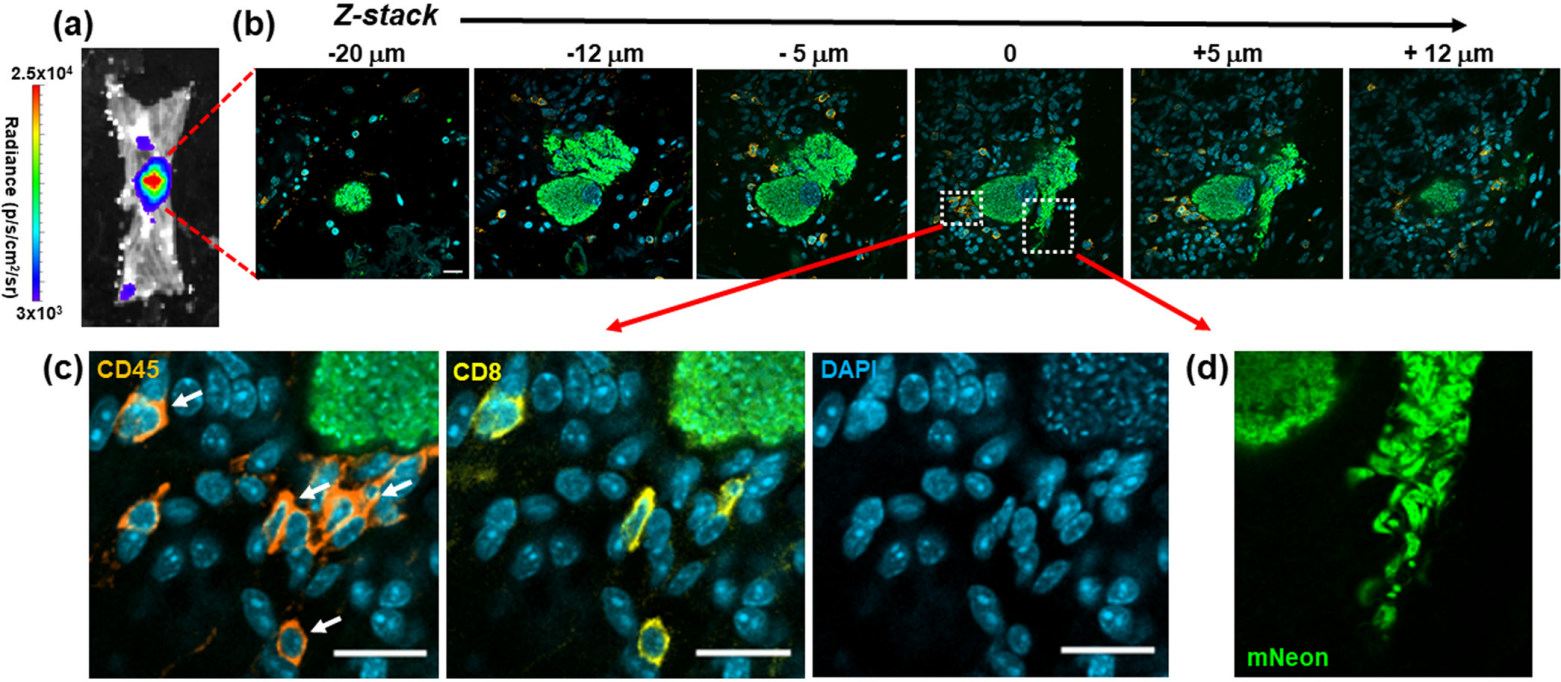
Incomplete recruitment of leukocytes to parasite nests allows progression of T. cruzi through the full intracellular infection cycle. (a) An intense bioluminescent focus in a chronic stage distal colon viewed by *ex vivo* imaging (Materials and Methods). Radiance (photons/s/cm^2^/sr) is shown on a linear-scale pseudocolor heat map. (b) Confocal imaging of the corresponding parasite nest showing representative serial Z-stack images taken along the depth of the infected cell. The *z* axis position relative to the center of the nest is indicated above each of the images. Parasite numbers (>1,000) were established from green fluorescence and the characteristic DAPI staining of the parasite kinetoplast DNA (the mitochondrial genome) (18) (blue). Infiltrating leukocytes (orange) were identified by staining with anti-CD45 antibodies (Materials and Methods). Bar = 20 μm. (c) Enlarged images of a small cluster of infiltrating CD45^+^ (orange) and CD8^+^ (yellow) cells in close vicinity to the nest. White arrows indicate leukocytes corresponding to CD8^+^ T cells. (d) Egress of differentiated trypomastigotes into the extracellular environment. Data from the infected cell captured in these images were not included in Fig. 7, since the parasite burden was too great to determine numbers with precision.

Of 237 infected colonic cells detected in 13 animals, only 4 (∼1.7%) contained parasites that had clearly differentiated into flagellated trypomastigotes, the life cycle stage that disseminates the infection via reinvasion of other host cells or via transmission to the blood-sucking triatomine insect vector. Of these, three contained large numbers of parasites (>1,000), while the fourth contained 128. In each case, the leukocyte densities in the local microenvironment were within a range similar to that in host cells where the infection was less mature, as judged by the number of intracellular amastigotes and their lack of differentiation into trypomastigotes. In the example shown (Fig. 8a and b), Z-stack imaging was used to serially section a large nest containing >1,000 parasites and shows mature trypomastigotes in the act of egress, despite the recruitment of a small number of CD45^+^ leukocytes, including CD8^+^ T cells (Fig. 8c and d). Therefore, for a small proportion of infected cells, including in some cases large and mature parasite pseudocysts, the host immune system either is not triggered locally by an infection, is too slow to respond, or is in some way suppressed. As a result, in the colon, it is possible for the entire cycle of parasite proliferation, differentiation, and egress to occur in the absence of effective intervention by a cellular immune response, at a level sufficient to allow prolongation of the chronic infection.

## DISCUSSION

Despite the generation of a vigorous and specific CD8^+^ T cell response (4, 14, 33, 34), T. cruzi infections in mice are rarely cleared to sterility, even in vaccinated animals. Instead, parasites persist predominantly in a small number of reservoir tissue sites, typically for the lifetime of the host (10). One possibility is that intermittent dissemination from these locations to less permissive organs, which include the heart, could promote repeated episodes of infection, resulting in localized inflammatory responses that contribute to disease pathology in a cumulative manner (35). Understanding why the immune system efficiently suppresses but fails to eliminate T. cruzi infections is one of the key challenges in Chagas disease research. Here, using tissue processing techniques that allow the immunological microenvironment of infection sites in the colon to be assessed at single-cell resolution, we demonstrate that CD45^+^ leukocytes, including both CD4^+^ and CD8^+^ T cells, are frequently recruited to chronic infection foci within a reservoir tissue. However, for a small subset of infected cells, effector cell recruitment is either absent or too slow to prevent completion of the intracellular cycle of parasite proliferation and differentiation to the motile trypomastigote stage (Fig. 8). Thus, chronic T. cruzi infections in the colon are characterized not by a generalized tissue-specific latency but by a dynamic equilibrium between host and pathogen.

T cell recruitment during T. cruzi infection is driven by secretion of chemokines from infected cells. For example, the CXCR3 ligands CXCL9 and CXCL10 have been implicated in cardiac infiltration (36). IFN-γ and tumor necrosis factor alpha (TNF-α) expression by antigen-specific CD8^+^ T cells (4), and subsequent expression of inducible nitric oxide synthase (iNOS) (37–39), potentially from recruited innate monocytes/macrophages or from somatic cells of the infected tissue, then increases the local concentration of reactive nitrogen species. In Chagas disease, the resulting inflammatory environment tightly controls the number of infected cells but can also act as the key driver of chronic immunopathology (7, 14, 40, 41). An important observation from our study is that the likelihood of T cell recruitment in the colon is not linked with the maturity of individual T. cruzi nests, as judged by the intracellular parasite load (Fig. 7). As a result, in some parasitized cells, differentiation to the flagellated trypomastigote form can occur without inducing infiltration of leukocytes in sufficient time to block a productive infection (Fig. 8).

The reasons why protective T cells are not recruited to a small subset of infection foci are unclear. Hypothesized mechanisms to account for T. cruzi immune evasion include a general absence of pathogen-associated molecular patterns (PAMPs) (42), cytokine-mediated inhibition of effector responses (10), insufficiently strong chemoattractant signaling in low-parasite-load settings (40), the extensive antigenic diversity expressed by the large families of *trans*-sialidase and mucin genes (14, 43, 44), and stress-induced cell cycle arrest and dormancy (19). However, none of these obviously corresponds with our observation that there is an apparent lack of association between the extent or longevity of an individual cellular infection and the magnitude of localized leukocyte recruitment (Fig. 7). Some highly infected host cells appear to be invisible to the immune system, whereas other much smaller nests trigger massive cellular infiltration. One explanation could be that a slowdown in the intracellular amastigote replication rate during chronic stage infections (12) contributes to reduced immune detection. In circumstances where the infected cell is in an area of the colon that is otherwise parasite free, this may be sufficient to permit completion of the initial replication cycle. However, after trypomastigote egress and host cell lysis, the resulting tissue disruption and production of damage-associated molecular patterns (DAMPs) could act to enhance leukocyte recruitment into the area, leading to the destruction of parasites that have reinvaded host cells in the vicinity of the initial infection. In contrast, trypomastigotes which migrate further from this DAMP-enriched locality may be able to establish a productive infection in the absence of rapid immune detection. Despite a diverse and complex antigenic repertoire, induction of the T cell response in draining lymph nodes is known to be highly focused (14), and once T cell recruitment has been triggered, parasite destruction can be initiated (Fig. 5d). Widespread parasite dormancy was not evident in the colon (12) and does not appear to be necessary for immune evasion in this tissue site.

Success or failure of the immune system in eliminating these rare chronic infection foci may be a largely stochastic process resulting from the dynamic interplay between the host and pathogen at a single-cell or tissue microdomain level. If parasites were able to universally suppress innate detection pathways, with concomitant reduction in localized chemokine output, this would have a negative impact on host survival and thus on long-term T. cruzi transmission. Conversely, if nests were always detected by the immune system before completion of the replication cycle, the parasite would risk host-wide elimination. The ability of T. cruzi to persist in some organs/tissues may therefore be dependent on the propensity, or otherwise, of these tissues to amplify the chemokine signals triggered by low-level infection, with a possible role for closely adjacent reinfections in the amplification process. In mice, there are strain-specific differences in the extent of such tissue restriction during chronic infections. This could have parallels in humans and account for the heterogeneous profile of disease progression. This requires much deeper analysis, because currently, there are insufficient data to rule out clonal antigenic variation as a mechanism contributing to perpetual immune evasion.

T. cruzi infection induces a high-titer polyclonal B cell/antibody response during the acute stage of infection, which, although delayed and initially unfocused (45), does contribute to parasite control and can protect against virulent infections. In the chronic stage, a role for the humoral response in suppressing the dissemination of persistent parasites is unresolved (10), and a key role for B cells has not been identified. Here, we show that in the absence of PBMCs, circulating antibodies, which in the short term are not profoundly affected by cyclophosphamide treatment (46) (Fig. 1d), are unable to compensate for T cell depletion and maintain tissue-specific repression of the parasite burden (Fig. 2 and 4d). If the humoral response does have a significant protective role during the chronic stage, for example, involving opsonization of the parasite through FcR-antibody binding, then this function could be lost on depletion of key cellular effectors. In addition, our results do not exclude the possibility that parasite-specific antibodies could act to limit infections at a systemic level, over a longer duration, perhaps by controlling trypomastigote numbers or restricting their spatial dissemination.

The central role of CD8^+^ T cells in suppressing T. cruzi infections is well established, and in various parasite-mouse strain combinations, depletion of circulating CD8^+^ T cells leads to partial recrudescence in specific organs (4, 5, 15, 34). In the experimental model outlined here, this relapse took place in skeletal muscle and skin (Fig. 2 and 3), although we cannot exclude the possibility that had the period of CD8^+^ depletion been extended, relapse, as inferred from the bioluminescence signal, would also have been identified at other sites. When we examined the effect of CD8^+^ T cell depletion at a cellular level in the colon, where tissue processing procedures allow systematic analysis, we found no significant increase in the number of infected cells, in contrast to the major rebound observed with cyclophosphamide-mediated reduction of the entire PBMC population (Fig. 1 and 4). Whether this was a result of less efficient depletion of CD8^+^ T cells at this site or was because the protective role is better covered by CD4^+^ T cells or innate populations will be an important question to address. A nonredundant function for CD4^+^ T cells is less well established in murine models of Chagas disease (47–49), although in humans with untreated HIV coinfections, parasites become easily detectable in the bloodstream and can result in central nervous system (CNS) pathology (50). Since depletion of either CD4^+^ or CD8^+^ T cells by themselves did not promote the level of systemic relapse observed with cyclophosphamide treatment over the time period analyzed (Fig. 2 and 3), our results therefore suggest either that both lymphocyte subtypes are able to contribute to suppression of chronic-stage infections in the colon or that innate monocytes/macrophages are able to provide a covering role during this period. The further development of tissue processing and imaging procedures applicable to other organs and tissues, to allow systematic analysis of chronic infections at single-cell resolution, will be an important step in extending these observations more widely.

If our findings in experimental mice are translatable to humans, this will have important implications for anti-T. cruzi vaccine development. Vaccines protect by presenting nontolerized antigens in the correct immunological context, to expand small numbers of antigen-specific naive T and B cells, which then generate a subpopulation of memory cells. The expanded memory populations then allow more rapid deployment of adaptive effectors on future contact with the pathogen. However, T. cruzi is able to persist indefinitely in hosts that already have expansive systemic populations of effective T cells. Unless vaccines can prevent parasites from accessing permissive sites after the initial infection or are able to enhance successful homing of adaptive effector cells, it will be difficult to achieve sterilizing immunity. Drug-cured infections can confer complete protection against rechallenge with a homologous strain, but with heterologous strains, despite the prevention of an acute-stage peak, the infection proceeds directly to a status that is analogous to the chronic stage in terms of parasite burden and tissue distribution (3). Therefore, it is likely that successful anti-T. cruzi vaccines will require an ability to eliminate parasites at the initial site of infection during the first intracellular replication cycle. This will be a considerable challenge.

## MATERIALS AND METHODS

### Mice and parasites.

All experiments were performed using female C3H/HeN mice, purchased from Charles River (UK). They were maintained in individually ventilated cages, under specific-pathogen-free conditions, with a 12-h light/dark cycle, and provided with food and water *ad libitum.* Research was carried out under UK Home Office project licenses PPL 70/8207 and P9AEE04E4, with approval of the LSHTM Animal Welfare and Ethical Review Board, and in accordance with the UK Animals (Scientific Procedures) Act 1986 (ASPA). The T. cruzi line CL Luc::mNeon, a derivative of the CL Brener strain (discrete typing unit TcVI), was used in all experiments. It had been genetically modified to express a bioluminescent-fluorescent fusion protein containing red-shifted luciferase and mNeonGreen fluorescent domains (27, 51). For infections, C3H/HeN mice, aged 6 to 8 weeks, were inoculated intraperitoneally (i.p.) with 1 × 10^3^ bloodstream trypomastigotes obtained from immunodeficient CB17-SCID mice, as described previously (30). Mice were then monitored by *in vivo* bioluminescence imaging (17), which indicated that they had transitioned to the chronic stage by 50 to 60 days postinfection. Experiments were performed when mice had been infected for at least 100 days.

### Suppression of the murine immune response.

General immunosuppression was achieved by injecting mice i.p. with cyclophosphamide (200 mg/kg) at 4-day intervals, up to a maximum of 3 injections, in accordance with animal welfare (17, 30). Circulating CD8^+^ T cells were depleted by i.p. injection of 400 μg of the YTS 169.4 monoclonal anti-CD8α (2BScientific), diluted in phosphate-buffered saline (PBS), at 4-day intervals, up to 4 times (Fig. 1C). The same regimen was applied for depletion of CD4^+^ T cells, using the GK1.5 monoclonal antibody (2BScientific).

### Tissue processing and imaging.

When mice were sacrificed, organs and tissues were removed and transferred to a petri dish in a standardized arrangement, soaked in 0.3 mg/mL d-luciferin in PBS, and examined by *ex vivo* bioluminescence imaging using the IVIS Spectrum system (Caliper Life Science) and LivingImage 4.7.2 software (52). The skin was removed from the carcass and, following subcutaneous adipose tissue removal, was placed fur down, soaked in 0.3 mg/mL d-luciferin, and imaged under the same conditions as the internal organs. The carcass was placed dorsal side up, soaked in 0.3 mg/mL d-luciferin, and imaged as described above.

Colonic muscularis walls were isolated by peeling away the mucosa, whole mounted as described previously (18), and then exhaustively searched for parasites (green fluorescence) with a Zeiss LSM880 confocal microscope. Small tissue sections (∼5 mm^2^) around parasite nests were excised from the whole mount by scalpel, washed twice in PBS, and incubated for 2 days in 1:300 primary antibody diluted in PBS–5% fetal calf serum–1% Triton X-100 at 4°C. Following 2 further washes in PBS, secondary antibody diluted 1:500 in the same blocking/permeabilizing solution was added to the tissue sections and incubated for 3 h at room temperature. Sections were then mounted in Vectashield containing the DNA stain DAPI and imaged by confocal microscopy. Colonic muscularis walls from naive age-matched mice were prepared similarly to controls, with and without the primary antibody.

For accurate determination of intracellular parasite and surrounding host cell numbers, tissue samples were imaged in 3 dimensions (Z-stacking), with the appropriate scan zoom setting (18). The Image Browser overlay function was used to add scale bars, and images were exported as TIF files to generate figures. Primary antibodies used were as follows: antiluciferase (G7451; Promega), anti-CD45 (Tonbo Biosciences; 30-F11), anti-CD3 (Abcam; ab11089), anti-CD4 (Abcam; ab25475), and anti-CD8 (Abcam; ab25478). The secondary antibodies were Invitrogen A-11055, Invitrogen A-21434, and Invitrogen A-11007.

### Flow cytometry.

At each time point, mice were placed in a “hot box” and left at 38°C for 10 min. They were then placed in a restrainer, and the lateral tail vein was punctured using a 0.5 M EDTA (pH 7.4)-soaked 21-gauge needle. A single drop of blood was transferred to a 2-mL tube, and 10 μL 0.5 M EDTA was added to prevent clotting. Each sample was then mixed with 400 μL ice-cold PBS, placed on 300 μL Histopaque 1083 (Sigma-Aldrich), and spun at 400 × *g* for 30 min in a microcentrifuge. The monocytic layer was aspirated using a pipette, mixed with 1 mL ice-cold PBS, pelleted, and resuspended in 200 μL flow cytometry buffer (PBS, 5% fetal bovine serum, 0.05% sodium azide), and 1 μL of the cocktail of conjugated antibodies was added (1:200 dilution in each case). After 1 h incubation in the dark, cells were pelleted and resuspended in 2% paraformaldehyde in PBS, followed by a further 45-min incubation in the dark. The stained fixed cells were then pelleted, resuspended in filtered flow cytometry buffer, and transferred to standard flow cytometry tubes. Samples were analyzed using a BD Bioscience LSRII flow cytometer, with plots created and analyzed in FlowJo V.10.6.1. The following antibodies were used: CD45 (Thermo Fisher; 30-F11; Super Bright 600), CD3 (Thermo Fisher; 17A2; FITC [fluorescein isothiocyanate]), CD4 (Thermo Fisher; RM4-5; eFluor 450), and CD8 (Thermo Fisher; SK1; Alexa Fluor 780).

### Anti-T. cruzi antibody enzyme-linked immunosorbent assay (ELISA).

Ninety-six-well plates were coated with sonicated T. cruzi CL Luc::mNeon trypomastigote lysate; 100 μL (0.5 μg) per well diluted in 15 mM Na_2_CO_3_–34.8 mM NaHCO_3_. The plates were incubated at 4°C overnight to allow antigen binding, washed three times with PBS–0.05% Tween 20, and then blocked with PBS–2% milk powder. Diluted murine serum samples, collected from each Histopaque separation, were further diluted to 1:1,600. These were aliquoted in triplicate (100 μL per well) and incubated for 1 h at 37°C. Horseradish peroxidase (HRP)-conjugated anti-mouse IgG secondary antibody (Abcam; ab99774) was then added (1:5,000; 100 μL per well), and the plates were incubated for a further 1 h. After the addition of HRP substrate (80 μL per well) (stabilized TMB [3,3′,5,5′-tetramethylbenzidine]; Life Technologies), the plates were incubated at room temperature in the dark for 5 min and read using a FLUOstar Omega plate reader (BMG Labtech), after the addition of 40 μL 1 M HCl.

### Testing for the effect of cyclophosphamide on T. cruzi growth.

A 96-well plate was seeded with 5,000 MA104 cells/well, and 18 h later these were infected with culture-derived T. cruzi CL Luc::mNeon trypomastigotes at a multiplicity of infection (MOI) of 5:1. Invasion was allowed to occur overnight, the wells were washed thoroughly with serum-free medium, and intracellular amastigotes allowed to proliferate for 24 h. Cyclophosphamide was then added up to a concentration of 200 μM. Three days later, the intensity of green fluorescence was recorded on a FLUOstar Omega plate reader, and the impact on parasite growth was assessed.

### Statistics.

Analyses were performed in GraphPad Prism v8.0. Standard deviation (SD) fold change in bioluminescence intensity was compared using a one-way analysis of variance (ANOVA) with Dunnett’s pairwise comparisons. Background cellularity and CD45^+^, CD4^+^, and CD8^+^ cutoffs were set as means plus 3 SD. Data sets were compared using a 2-sample *t* test with Welch correction. If data were not normally distributed, as assessed using a Shapiro-Wilk test, a Mann-Whitney rank sum test was used.
