# Supplementary material for: Incomplete Recruitment of Protective T Cells Is Associated with Trypanosoma cruzi Persistence in the Mouse Colon
Source: Infect Immun. 2022 Feb 17;90(2):e00382-21. doi: 10.1128/iai.00382-21 (PMC8853677; doi:10.1128/iai.00382-21)
Supplement: Supplemental file 1 — Supplemental material. Download iai.00382-21-s0001.pdf, PDF file, 0.5 MB [file iai.00382-21-s0001.pdf]

Incomplete recruitment of protective T cells is associated with *Trypanosoma cruzi* persistence in the mouse colon

Alexander I. Ward, Michael D. Lewis, Martin C. Taylor and John M. Kelly

Supplementary Figures

FIG S1 Assessment of the effect of cyclophosphamide on the intracellular growth of *T. cruzi*. MA 104 cells (an African green monkey fetal kidney line) seeded into a 96-well microtitre plate were infected with *T. cruzi* CL Luc::mNeon trypomastigotes (Material and Methods). Intracellular amastigotes were allowed to proliferate for 24 hours, and cyclophosphamide was then added at a range of concentrations up to 200 μM. 3 days later, the effect on growth relative to untreated controls was inferred from the intensity of green fluorescence recorded on a FLUOstar Omega plate reader. Data points are plotted with the standard error (n=8)

FIG S2 Flow cytometry gating strategy. (a) PBMCs isolated in the black oval based on forward (FSC) and side (SSC) scatter spectral properties. (b) Singlets isolated. (c) Population staining +ve with anti-CD45 antibody. (d) $CD45^+$ population separated by CD3 positivity. (e) Both $CD3^+$ and $CD3^-$ populations separated by CD4 and CD8 markers.

FIG S3 Establishing the extent of $CD8^+$ T cell recruitment to infection foci using 3-dimensional serial Z-stack confocal imaging. (a) A parasite nest detected in the whole mounted colonic gut wall of a mouse chronically infected with *T. cruzi* CL Luc::mNeon (Materials and Methods). Parasites, green; DNA, blue (DAPI); $CD8^+$ T cells, yellow (stained with antibody prior to mounting). The area selected for Z-stack imaging is identified by a 200 μm diameter circle, centred on the parasite nest. (b) The local density of $CD8^+$ host cells was determined by counting the number of stained cells (yellow) in a series of Z-stack images acquired with a Zeiss LSM880 confocal microscope from 5 μm above and below the centre of the parasite nest on the Z-axis,

a cylinder volume of 314 μm^3. Any cells that fell within the 200 μm diameter circle were included. The number of $CD8^+$ T cells was calculated to be 44.

FIG S4 The longitudinal and transverse smooth muscle layers of the colon are largely devoid of $CD45^+$ leukocytes in non-infected C3H/HeN mice. (a) Serial Z-stack images of a whole mounted colonic gut wall from an age-matched non-infected C3H/HeN mouse. DNA, blue (DAPI); $CD45^+$, orange. Scale bars=20 μm. The images correspond to the cross-sectional regions of the colon indicated in the schematic (1-5). $CD45^+$ cells can be readily detected in the sub-mucosal layer (inset). (b) Rare example of a $CD45^+$ cell within the longitudinal and transverse smooth muscle layers. A 200 μm diameter circle is superimposed on the image.

Fig. S1

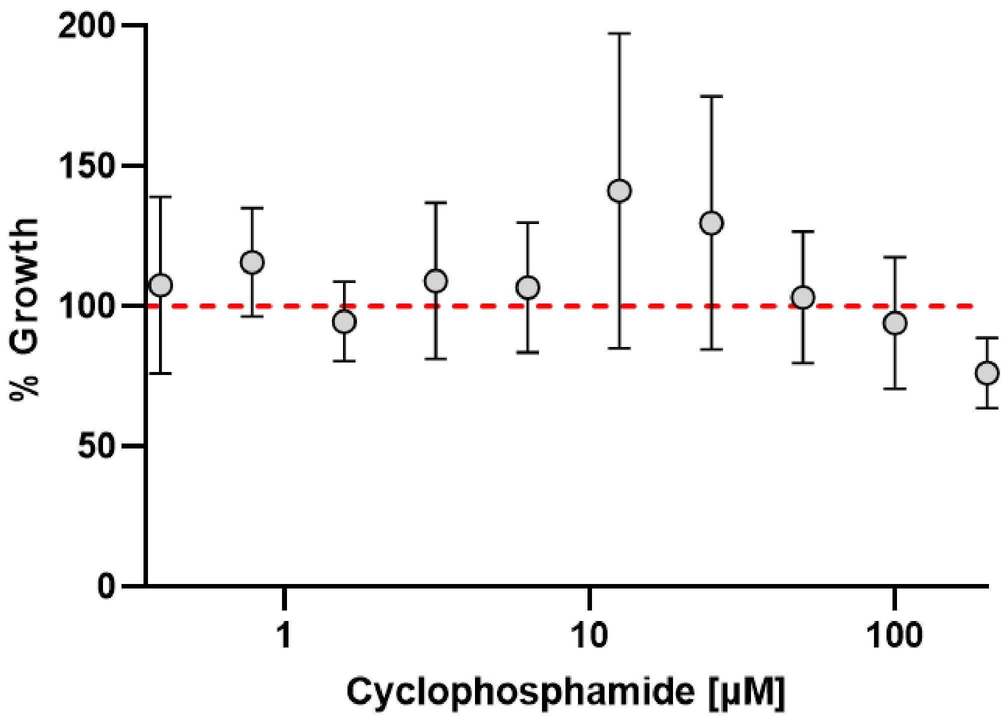

Fig. S2

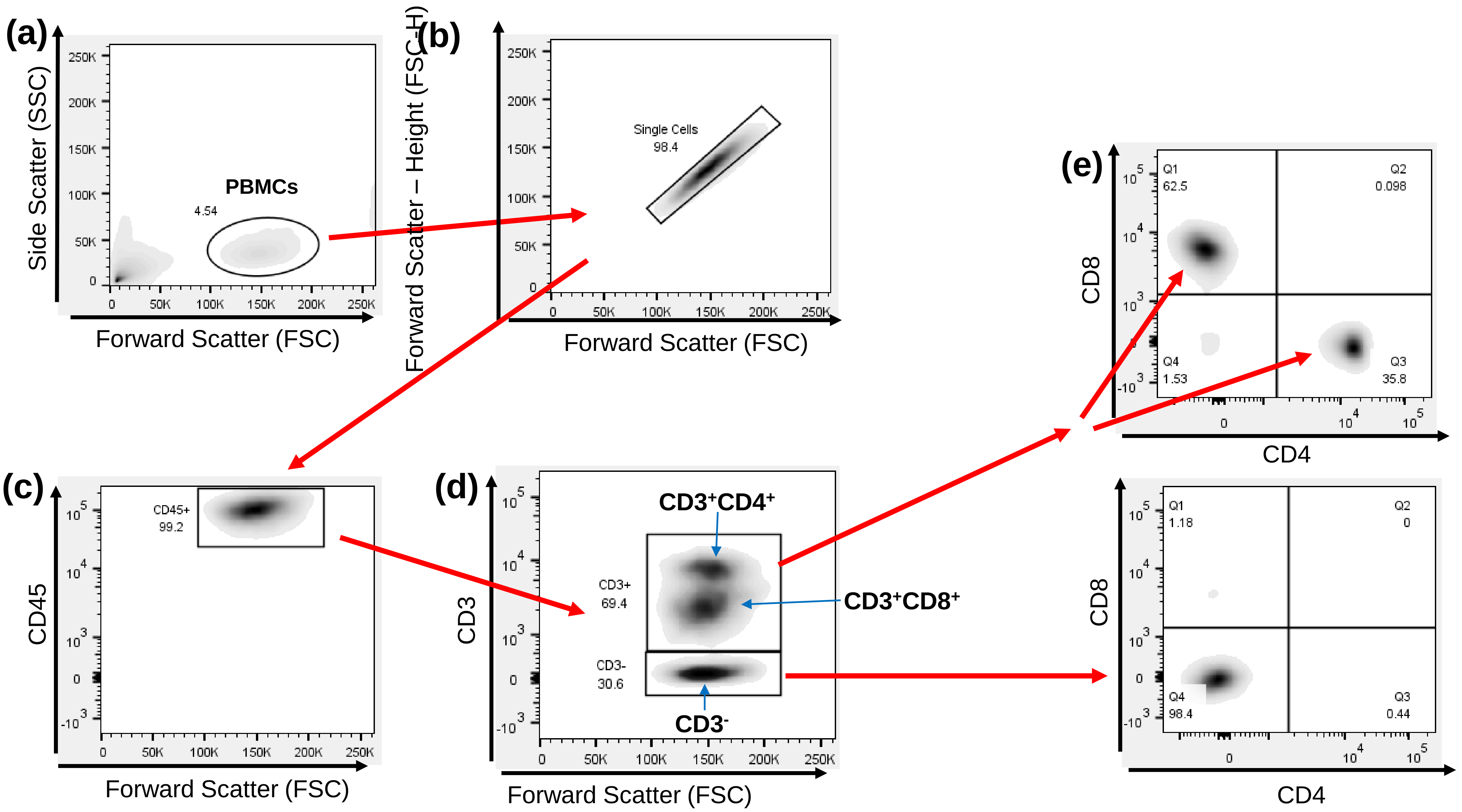
(a)
Side Scatter (SSC)
PBMCs
4.54
Forward Scatter (FSC)
(b)
Forward Scatter – Height (FSC-H)
Single Cells
98.4
Forward Scatter (FSC)
(c)
CD45
CD45+
99.2
Forward Scatter (FSC)
(d)
CD3
CD3+CD4+
CD3+
69.4
CD3+CD8+
CD3-
30.6
CD3-
Forward Scatter (FSC)
(e)
CD8
Q1
62.5
Q2
0.098
Q4
1.53
Q3
35.8
CD4
CD8
Q1
1.18
Q2
0
Q4
98.4
Q3
0.44
CD4

Fig. S3

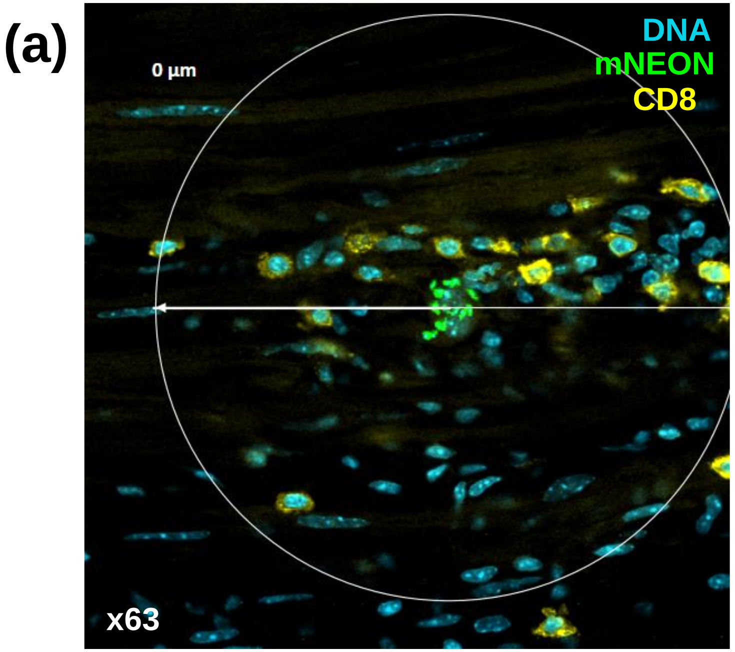
(a)
0 µm
DNA
mNEON
CD8
x63

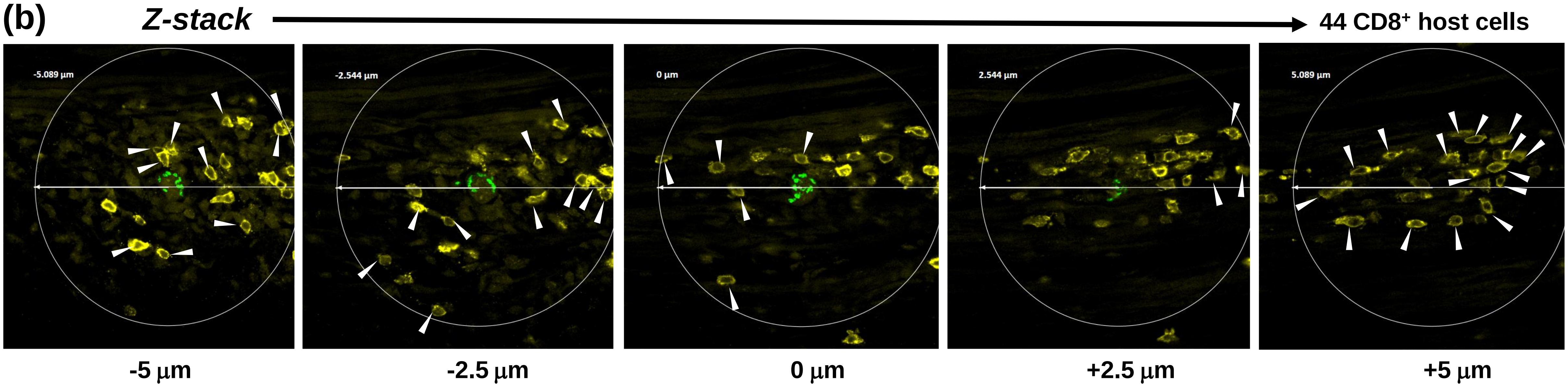
(b)
Z-stack
44 CD8⁺ host cells
-5.089 µm
-2.544 µm
0 µm
2.544 µm
5.089 µm
-5 µm
-2.5 µm
0 µm
+2.5 µm
+5 µm

Fig. S4

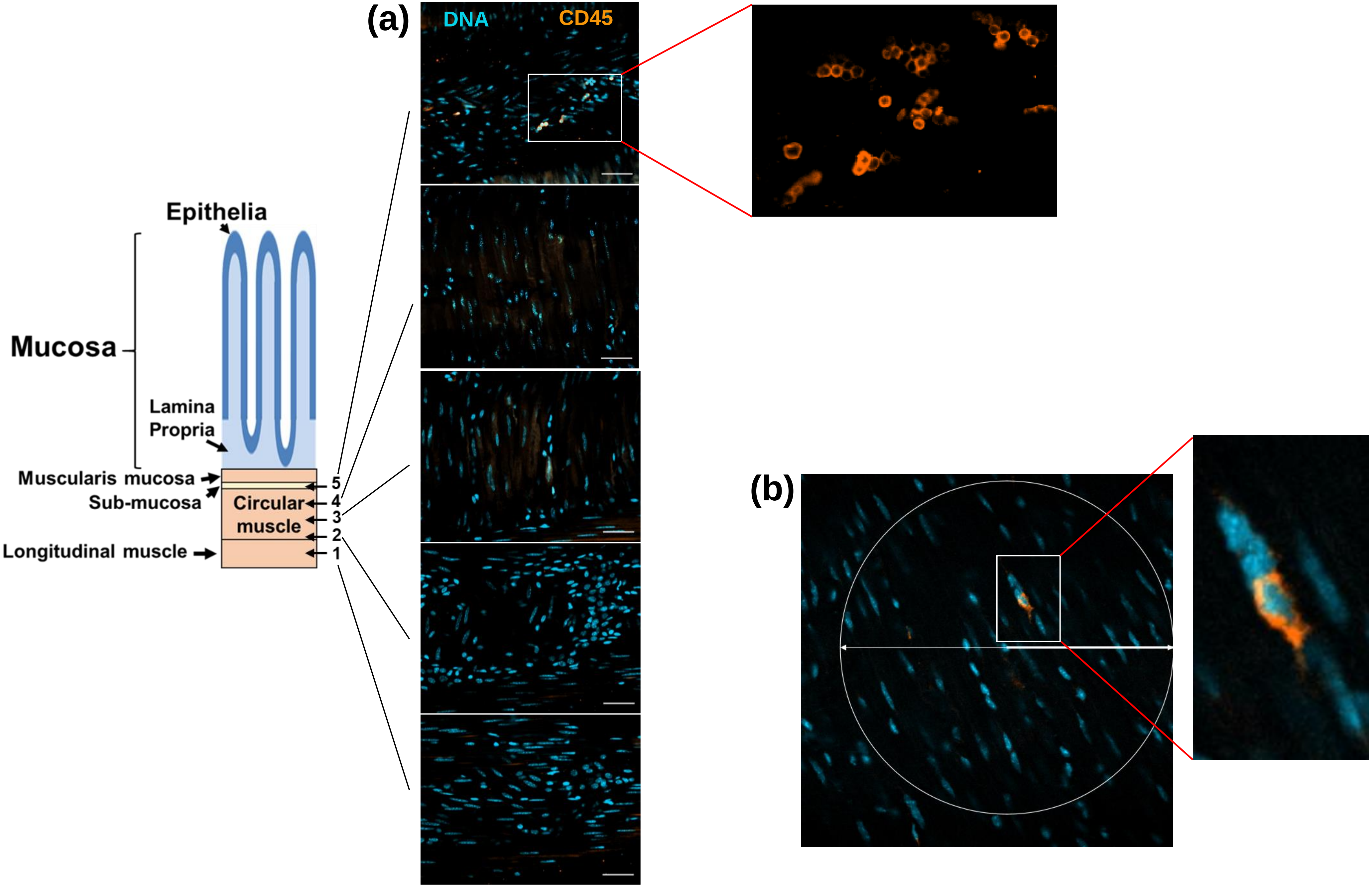
(a)
DNA
CD45
Epithelia
Mucosa
Lamina
Propria
Muscularis mucosa
Sub-mucosa
Circular
muscle
Longitudinal muscle
5
4
3
2
1
(b)
